# An international survey on anastomotic stricture management after esophageal atresia repair: considerations and advisory statements

**DOI:** 10.1007/s00464-020-07844-6

**Published:** 2020-08-03

**Authors:** Chantal A. ten Kate, Renato Tambucci, John Vlot, Manon C. W. Spaander, Frederic Gottrand, Rene M. H. Wijnen, Luigi Dall’Oglio

**Affiliations:** 1 Department of Pediatric Surgery, Erasmus University Medical Center - Sophia Children’s Hospital, Rotterdam, The Netherlands; 2 Digestive Endoscopy and Surgery Unit, Bambino Gesù Children’s Hospital - Research Institute IRCCS, Piazza S. Onofrio 4, 00165 Rome, Italy; 3 Department of Gastroenterology and Hepatology, Erasmus University Medical Center, Rotterdam, The Netherlands; 4 Department of Pediatric Gastroenterology Hepatology and Nutrition, CHU Lille, Univ. Lille, Lille, France

**Keywords:** Esophageal atresia, Anastomotic strictures, Dilatation management

## Abstract

**Background:**

Endoscopic dilatation is the first-line treatment of stricture formation after esophageal atresia (EA) repair. However, there is no consensus on how to perform these dilatation procedures which may lead to a large variation between centers, countries and doctor’s experience. This is the first cross-sectional study to provide an overview on differences in endoscopic dilatation treatment of pediatric anastomotic strictures worldwide.

**Methods:**

An online questionnaire was sent to members of five pediatric medical networks, experienced in treating anastomotic strictures in children with EA. The main outcome was the difference in endoscopic dilatation procedures in various centers worldwide, including technical details, dilatation approach (routine or only in symptomatic patients), and adjuvant treatment options. Descriptive statistics were performed with SPSS.

**Results:**

Responses from 115 centers from 32 countries worldwide were analyzed. The preferred approach was balloon dilatation (68%) with a guidewire (66%), performed by a pediatric gastroenterologist (*n* = 103) or pediatric surgeon (*n* = 48) in symptomatic patients (68%). In most centers, hydrostatic pressure was used for balloon dilatation. The insufflation duration was standardized in 59 centers with a median duration of 60 (range 5–300) seconds. The preferred first-line adjunctive treatments in case of recurrent strictures were intralesional steroids and topical mitomycin C, in respectively 47% and 31% of the centers.

**Conclusions:**

We found a large variation in stricture management in children with EA, which confirms the current lack of consensus. International networks for rare diseases are required for harmonizing and comparing the procedures, for which we give several suggestions.

**Electronic supplementary material:**

The online version of this article (10.1007/s00464-020-07844-6) contains supplementary material, which is available to authorized users.

Despite improved treatment strategies, up to 60% of children with esophageal atresia (EA) develop an anastomotic stricture after surgical correction, mostly in the first year of life [1]. Based on a recent guideline for the management of complications in children with EA of the European Society for Pediatric Gastroenterology, Hepatology and Nutrition (ESPGHAN) and North American Society for Pediatric Gastroenterology, Hepatology and Nutrition (NASPGHAN), the first-line treatment for anastomotic strictures is endoscopic dilatation under general anesthesia [2].

Currently, there is no consensus on how dilatation should be performed [3]. Two different methods of endoscopic dilatation are used: balloon dilatation and semi-rigid dilatation, i.e., bougienage. The primary goals of esophageal dilatation are symptom relief, maintenance of age-appropriate oral nutrition, and reduction of pulmonary aspiration risk. Balloon dilatation applies a radial force over the entire length of the esophageal stricture, while bougies generate shearing axial forces as they pass across the stenosis (see Fig. 1) [4]. Currently, there are no randomized controlled trials comparing the efficacy and safety of balloon dilatation and bougienage for the treatment of anastomotic strictures in children with EA. Data from published studies on pediatric esophageal strictures of varying etiology show conflicting results [5–7]. A recent meta-analysis included five randomized controlled trials that have compared the two techniques in adults with benign esophageal strictures; the results indicated no significant differences in efficacy and safety [8–13].

**Fig. 1:**
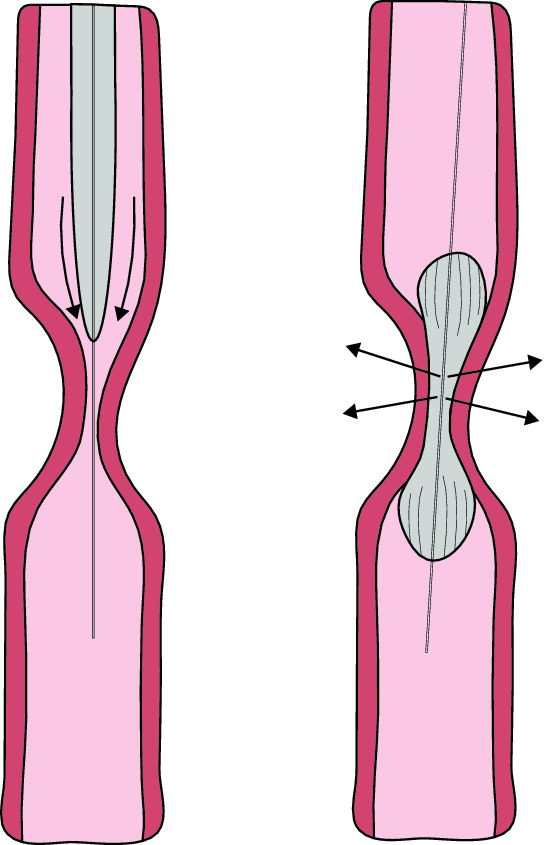
Bougienage (left) creates axial forces; balloon dilatation (right) creates mainly radial forces, as shown by the arrows

Due to the lack of strong evidence, the choice of dilatation method is currently based on the center’s and operator’s experience and preference. To come to consensus and guidelines, it is important to know how management dilatation strategies are currently applied in different centers. Therefore, we performed a survey study with the aim to provide an overview on differences in endoscopic dilatation treatment of pediatric anastomotic strictures worldwide.

## Participants and methods

We conducted a cross-sectional survey study from November 2018 up to and including March 2019. The study was approved by the institutional review board of the Erasmus University Medical Center Rotterdam, the Netherlands (MEC-2018-1463). A free-access online questionnaire (LimeSurvey GmbH version 2.06lts, Hamburg, Germany) consisting of 38 questions in the English language was distributed via e-mail and newsletters to all members of the ESPGHAN EA Working Group, the NASPGHAN, the Australian Society of Paediatric Gastroenterology Hepatology and Nutrition (AuSPGHAN), the European Pediatric Surgeons’ Association (EUPSA), and the International Network of Esophageal Atresia (INoEA). Distribution via other pediatric associations was not possible since we were not able to initiate collaboration. Members of these associations were asked to forward the online questionnaire to colleagues in the field. A reminder was sent one month after initial contact. Participants could be of any specialty, as long as they had experience in the treatment of anastomotic strictures in children with EA. In case of multiple responses per center, responses were pooled to an average. If no average could be calculated, we included the most comprehensive answer. If less than 80% of the questions was answered, the questionnaire was excluded from the analysis.

A draft questionnaire was made based on both literature and expert opinion from the ESPGHAN EA Working Group meeting in Geneva on May 2018. This draft was reviewed and approved by the EUPSA Network Office. All members of the research team were invited to comment on the draft version; comments were accounted for in the final version.

In brief, the survey questions concerned: the center the respondent was working at, the number of dilatation procedures performed in the center per month, the preferred dilatation technique (balloon dilatation or bougienage) and the use of alternative or adjuvant treatment options for recurrent strictures. The complete questionnaire can be found in Supplementary File 1.

The main outcome was the difference in endoscopic dilatation treatment of stricture formation after EA repair in various centers worldwide, including technical details (e.g., insufflation material and duration, use of a guidewire or fluoroscope), dilatation approach and adjuvant treatment options. Descriptive statistics were applied to the data. Answers were mainly categorical. Data are represented as number (%). All data was analyzed using SPSS V.24.0 (IBM, Chicago, Illinois, USA). The CHERRIES (Checklist for Reporting Results of Internet E-Surveys) checklist was used as a reporting framework [14].

## Results

### Response

**Fig. 2:**
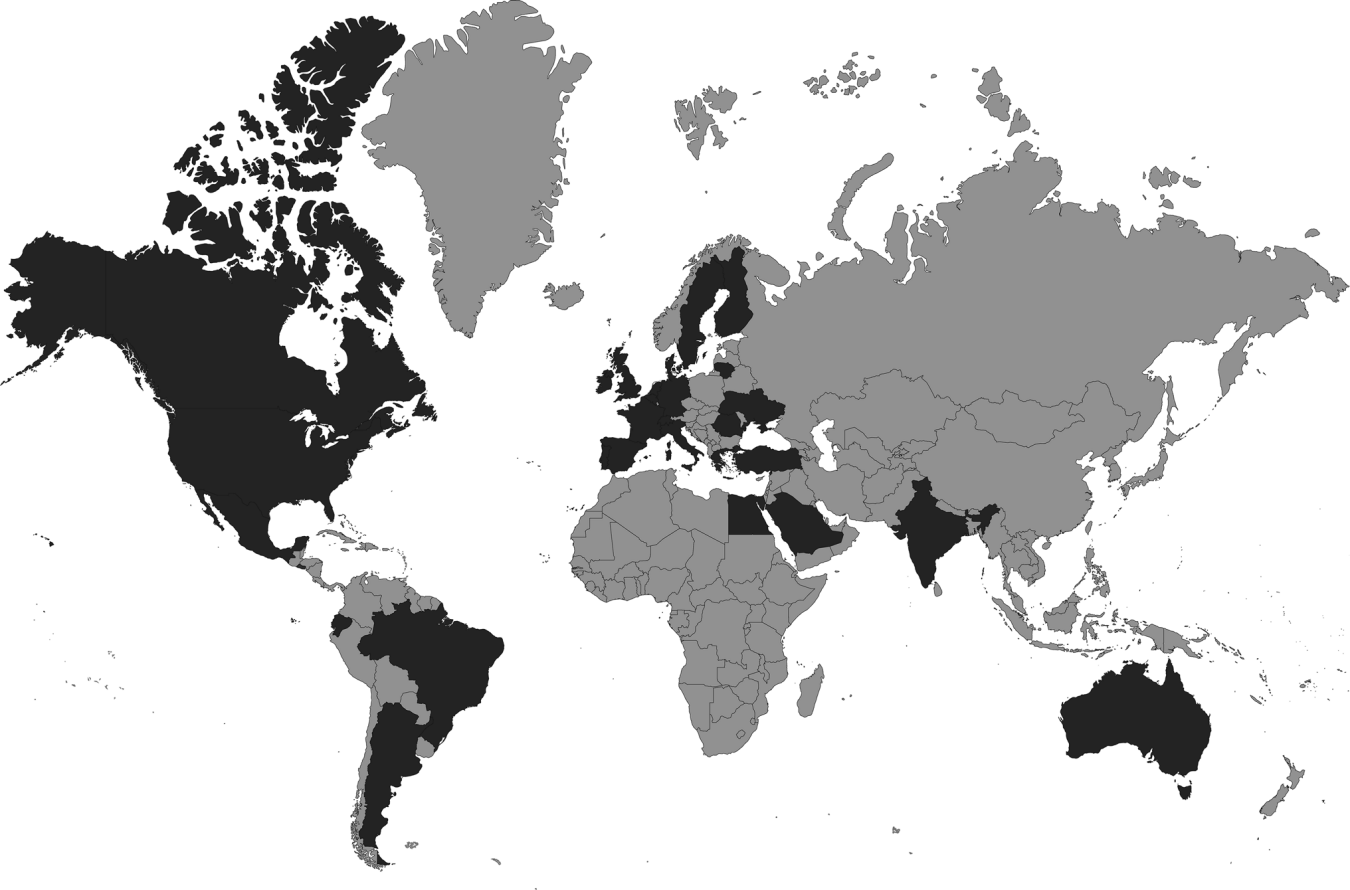
Participating centers (*n* = 115 in dark grey) in 32 countries spread over six continents. Figure created with: https://www.amcharts.com/visited_countries/

**Fig. 3:**
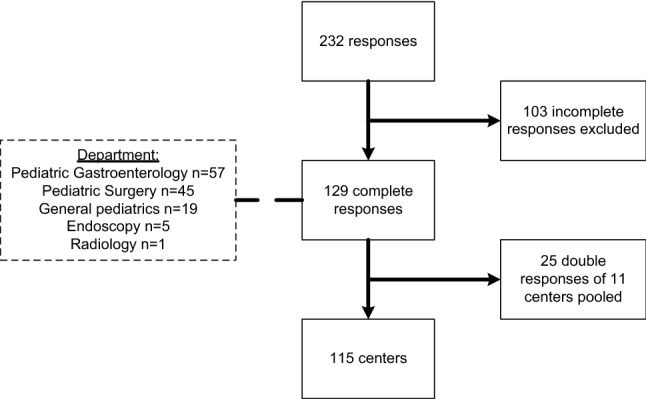
Flowchart of the responses

In total, 232 questionnaires were filled out. Since the initial recipients had been asked to forward the questionnaire to other clinicians, a response rate could not be calculated. Responses came from 32 countries worldwide (see Fig. 2). Sixty percent of the responses came from European countries, 24.3% from North American countries and 15.7% from other continents. After excluding 103 incomplete questionnaires with < 80% of the questions answered and pooling 25 duplicate responses of 11 centers, data from 115 centers remained for analysis (see Fig. 3). The majority of the centers (87.8%) were academic centers. The majority of the responses came from departments of Pediatric Gastroenterology (*n* = 57) and Pediatric Surgery (*n* = 45).

### Physicians performing endoscopies

The majority of the centers performed 10–30 pediatric endoscopies per month, but less than five pediatric esophageal dilatation procedures per month. Half of the centers performed less than three dilatation procedures for anastomotic strictures in patients with EA per month. All center demographics are listed in Table 1.

**Table 1:** Demographics of the participating centers (*n* = 115)

Characteristic	n (%)
Continent	
Europe	69 (60)
North America	28 (24.3)
South America	6 (5.2)
Africa	5 (4.3)
Asia	4 (3.5)
Oceania	3 (2.6)
Total number of pediatric upper endoscopies^a^ (per month)	
< 10	8 (7.0)
10–30	51 (44.3)
31–50	25 (21.7)
51–70	9 (7.8)
> 70	22 (19.1)
Number of pediatric esophageal dilatation procedures^b^ (per month)	
< 5	61 (53.0)
5–10	33 (28.7)
11–15	6 (5.2)
16–20	2 (1.7)
> 20	5 (4.3)
Unknown	8 (7.0)
Number of patients with EA < 18 years under follow-up	
< 20	27 (23.5)
20–40–	34 (29.6)
41–60	14 (12.2)
61–80	12 (10.4)
81–100	4 (3.5)
> 100	21 (18.3)
Unknown	3 (2.6)
Number of dilatation procedures for anastomotic strictures in patients with EA (per month)	
< 3	60 (52.2)
3–5	34 (29.6)
6–7	9 (7.8)
8–10	3 (2.6)
> 10	3 (2.6)
Unknown	6 (5.2)

*EA* esophageal atresia

^a^Both diagnostic and therapeutic, in all pediatric patients

^b^In all pediatric patients

Endoscopies were most frequently performed by pediatric gastroenterologists (*n* = 103) and pediatric surgeons (*n* = 48), and less often by adult gastroenterologists (*n* = 24) or adult surgeons (*n* = 12). Two centers had employed a specialized pediatric endoscopist. In 85 of the 101 academic centers (84.2%) trainees performed endoscopies as well.

### Approach for anastomotic strictures

Seventy-eight centers (68.4%) performed selective dilatations, meaning they performed a dilatation procedure only in symptomatic patients. In 36 centers (31.6%) routine dilatations were performed to prevent symptoms to occur; these centers planned subsequent dilatations in advance.

Balloon dilatation was the preferred technique to treat anastomotic strictures in patients with EA in 78 centers (67.8%). Twenty centers (17.4%) preferred semi-rigid dilatation or bougienage; seventeen centers (14.8%) applied both techniques.

**Fig. 4:**
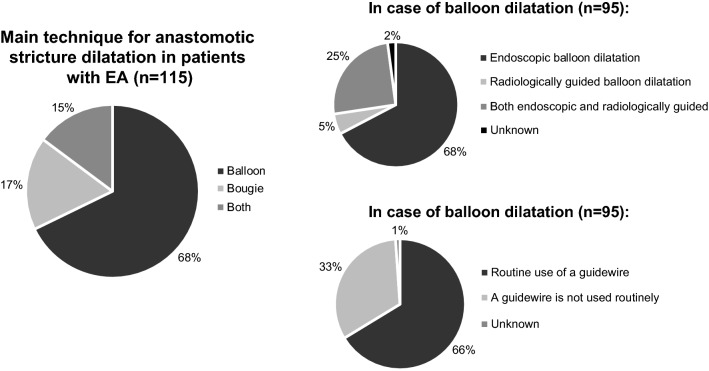
The mainly used techniques to manage esophageal anastomotic strictures in patients with esophageal atresia (EA)

In total, balloon dilatation was applied in 95 centers—regardless if it was the preferred technique or not. In 88 of those 95 centers, this was done endoscopically. Twenty-nine of those 95 centers used a radiologically guided approach; sixty-three centers routinely used a guidewire. See also Fig. 4.

### Balloon and semi-rigid dilatation

For balloon dilatation, the Controlled Radial Expansion (CRE) balloon dilatator from Boston Scientific™ was used most often (*n* = 66). Alternatively, twenty-two centers used the Rigiflex dilatator (Boston Scientific™), eight centers used the Ultra-Thin Diamond dilatator (Boston Scientific™), and six centers used the Maxforce (Boston Scientific™). The Hercules dilatator (Cook Medical®) and the Percutaneous Transluminal Valvuloplasty Balloon Catheter (VACS®, B. Braun Medical B.V.) were the least used, in two and one centers, respectively.

Forty-nine centers used water or 0.9% natrium chloride to insufflate the balloon, forty-six centers used contrast fluid, and 16 centers used air. Some centers used multiple types of insufflation material since they also used multiple types of dilators. The design of the questionnaire did not permit correlating the type of insufflation with each type of dilatator. The insufflation duration was standardized in 59 centers (51.3%). Across the centers, the median insufflation duration was 60 s (range 5–300).

For bougienage, twenty-seven centers used the Savary-Gilliard dilatator (Cook Medical®). Other semi-rigid dilatators used were the Tucker dilatator (Teleflex®) in seven centers, the Maloney dilatator (Pillings®) in seven centers, the American Dilatation System dilatator (Bard™) in three centers, the Rehbein dilatator (Rush®) in one center, and the Hurst dilatator (Pillings®) in one center.

None of the centers had a well-designed protocol to determine which diameter of the dilatator should be used nor to which diameter should be dilated. Eleven centers had set choices, but with specifications like “based on the age of the patient”, “if resistance is felt” and “we progress depending on the situation”.

### Recurrent and refractory strictures

Most centers had different adjuvant treatment options available for recurrent and refractory anastomotic strictures. Local injection with steroids was available in 77 centers, topical application of mitomycin in 66, esophageal stenting in 41, and incisional therapy in 30 centers. In 10 centers, other treatment options were available to treat refractory strictures: four centers would prescribe anti-reflux medication or advise fundoplication surgery; four centers would re-operate and perform a resection with a new anastomosis; one center would prescribe budesonide oral gel; and one center would inject vitamin B into the stenosis. Indwelling balloon catheter is a method described in literature [15] but none of the participating centers in this survey mentioned to practice this option. Overall, the majority preferred local injection of steroids (56 centers, 47.1%) or topical application of mitomycin C (37 centers, 31.1%) as first-line adjuvant treatment for a refractory stricture.

## Discussion

The aim of this survey study was to provide insight in the differences in endoscopic dilatation methods used for stricture formation after EA repair worldwide. The results show a great variation in the way dilatation procedures are performed. Overall, the preferred technique was balloon dilatation with a selective approach; i.e., performed only in symptomatic patients.

According to this survey, pediatric gastroenterologists perform the majority of the endoscopies, followed by pediatric surgeons. The literature contains no studies comparing the success rates of dilatation of anastomotic strictures by different specialists. Generally, it is acknowledged that these procedures are the safest and most effective when performed by a skilled and experienced operator [2]. In this age of patient-centered care, one could raise the question of whether there is a place for adult specialists in the treatment of pediatric patients with rare diseases. In this regard, it is our opinion that dilatation of anastomotic strictures in children with EA should be executed by a pediatric gastroenterologist or pediatric surgeon with experience in the management of this population. Smaller centers, where a pediatric gastroenterologist or pediatric surgeon is not available, should refer these children to a nearby expert center. The fact that almost 90% of the centers in this survey were academic centers indicates that this may already be common practice.

The majority of the surveyed centers preferred a selective approach; i.e., dilate an anastomotic stricture only in symptomatic patients. The idea behind this “wait and see” approach is to reduce the number of dilatations, and consequently the exposure to anesthesia and possible complications of a dilatation. Anesthetic exposure at young age is associated with gross motor problems, learning disabilities, behavioral problems, and developmental disorders [16–19]. On the other hand, proponents of routine dilatations advocate that complex strictures—and therefore long-term functional problems—can be prevented by preserving a minimum diameter.

Two retrospective studies have compared selective dilatations with routine dilatations [20, 21]. Selective dilatations were associated with significantly fewer dilatations and a significantly shorter hospital stay than routine dilatations. Occurrences of dysphagia, respiratory complaints, and bolus obstruction did not significantly differ between the two approaches.

The ESPGHAN-NASPGHAN guideline recommends close follow-up during the first 2 years of life, with special attention to the first introduction of solid food. This holds as well for patients with a long gap EA and/or postoperative anastomotic leakage, which are risk factors for stricture development [1]. However, since there is no evidence supporting the more invasive strategy of routine dilatations, the expert opinion in this guideline states that the presence of an anastomotic stricture should be excluded and treated in symptomatic children only [2].

Two-third of the centers preferred balloon dilatation to bougienage to manage anastomotic strictures in patients with EA. Where bougienage as therapy for esophageal strictures has been reported for almost 200 years, balloon dilatation—introduced in 1981—is relatively new [22, 23]. As mentioned earlier, the main difference between the techniques is the type of forces applied to the stricture. Balloon dilatators create radial forces and allow for a consistent treatment when the balloon is insufflated according to a standardized protocol. Bougies exert axial forces, which the operator can adjust as he or she considers necessary.

Literature comparing the two techniques is scarce and with divergent results. Some retrospective studies in both children and adults with a variety of esophageal strictures reported no differences in safety, effectiveness, and complications [11, 24, 25]. Other studies in children found favorable results for balloon dilatation. For example, significantly fewer dilatations required [5] and significantly fewer technical failures, defined as no passage possible through the stenosis [6]. On the other hand, a study in 47 children with congenital esophageal stenosis found a significantly lower perforation rate for bougienage than for balloon dilatation [7]. Two randomized controlled trials in adults with dysphagia due to benign esophageal strictures found no differences between the two techniques except less discomfort during balloon dilatation (*p* < 0.05)—which in adults usually is performed in awake or lightly sedated state [8, 9]. Prospective comparative studies in children are lacking.

The most used balloon dilatator in the surveyed centers was the CRE balloon dilatator from Boston Scientific™. All balloon dilatators reported in the survey were through-the-scope dilatators, enabling direct vision during the procedure when being used with a medium-sized scope like for example the Olympus Q180, which has an instrumental channel with a diameter of 2.8 mm. These dilatators are designed to pass the scope without the use of a guidewire. A guidewire is still included; however, in the CRE and Rigiflex dilatators, so they can also be used in combination with a small-sized scope (for example the Olympus GIF-XP190 with an instrumental channel with a diameter of 2.2 mm), separately through the nose or mouth. The CRE and Hercules dilatators are ‘3-stage dilatators’, designed to produce three distinct diameters based on the pressure caused by insufflation. The VACS dilatator is actually designed for heart surgery; its smallest diameter is 4 mm. This makes this dilatator very suitable for severe strictures with a small lumen.

The Savary-Gilliard dilatator (Cook Medical®) was used most frequently for bougienage. This is a wire-guided bougie dilatator with a long tapered tip and a radiopaque marking at the base of the taper. Other wire-guided bougies are American Dilatation System, Tucker, and Rehbein dilatators. In contrast to Savary-Gilliard dilatators, American Dilatation System dilatators have a shorter taper but total radiopacity. Tucker and Rehbein dilatators are small silicone bougies with a tapered end at each side, and can only be used in gastrostomized cases. Hurst and Maloney dilatators are the only bougies that do not accommodate a guidewire. These tungsten-filled dilators are helped by gravity. Hurst dilatators have a blunt tip; Maloney dilatators have a more tapered tip.

The literature contains no studies comparing the different types of dilatators [26]. Currently available studies on dilatation management hardly—or not at all—report the type of dilatator. One could argue that the type of dilatator does not matter as long as it is manipulated by an experienced operator. Nevertheless, it would be good to standardize the application and technical details of both methods in a guideline, especially for rare conditions like anastomotic strictures in children with EA.

Regarding the technical details, it appeared that 29 out of the 95 centers that preferred balloon dilatation used a radiologically guided approach, in line with the finding that most of the centers used water, natrium chloride, or contrast fluid to insufflate the balloon. The manufacturers of the CRE and Maxforce dilatators recommend to insufflate the balloon with water. For the Hercules and VACS dilatators, the manufacturers instruct hydrostatic pressure, which can be either water, saline or contrast fluid. Insufflation with air is advised for the Rigiflex dilatator. We could not find an instruction manual for the Ultra-Thin Diamond dilatator.

As we know from basic physics, gases are easier to compress than are fluids. Hydrostatic pressure is safest: in case of a balloon rupture, air would create a catastrophic burst [27]. Although evidence on this issue is lacking, we advise to only insufflate balloons with fluids (i.e., water, natrium chloride or contrast) and to use a dilatation system that supports hydrostatic pressure.

The insufflation procedure has been standardized in a protocol in half of the participating centers, albeit with a wide range of the dilatation duration, from 5 to 300 s. Although a small randomized controlled trial in 20 adults suggested that insufflation for 10 s is as effective as insufflation for 2 min [28], we still argue—on the bases of our experience—for a standardized duration of one minute per dilatation to a certain diameter. Standardization provides the opportunity to evaluate the efficacy of this duration, and adjust the duration if necessary.

The optimum diameter for dilatation is difficult to determine, as is also apparent in this survey. None of the centers had a protocol in place to make this decision. Combining the results of this survey with the literature, we conclude that currently the most common method to determine the diameter of the healthy esophagus is the “rule of thumb”. This means that the diameter of the thumb equals the diameter of the esophagus. A recent study found a strong correlation between body weight and the diameter of the esophagus [29]. This is a recent finding which needs further investigation; for now, we support application of the “rule of thumb”.

With regard to recurrent strictures, most of the participating centers preferred local steroid injection or topical mitomycin application as first-line adjuvant treatment. Although promising results have been published for both methods, evidence in children remains scarce [2, 30–33].

We propose to leave the application of adjuvant treatments to expert centers only, which can decide on the proper treatment based on the patient’s characteristics, the stricture and the operator’s experience. In addition, centralizing the management of refractory strictures would increase patient numbers, thereby raising the possibilities for comparative research. It has already been acknowledged that centralization and introducing minimal volume standards for referral centers can lead to an improvement in outcome [34, 35]. In a recent consensus conference of the European Reference Network on Rare Inherited and Congenital Anomalies (ERNICA), a minimum caseload of five new patients with EA per year was defined as a requirement of an expert center [36]. Based on our expert opinion, we therefore propose that a center should perform minimally 10 dilatations in patients with EA per year, and otherwise refer their patients to an expert center. Although 10 dilatations per year is still a low frequency, at least this will avoid incidental dilatation procedures. This volume-based strategy should of course be evaluated to see if caseload influences the outcomes and complication rates, especially for recurrent strictures.

To our knowledge, this is the first international survey on dilatation management in anastomotic strictures after EA repair. An earlier EUPSA survey addressed the surgical treatment of EA in general, but did not pay attention to the management of strictures [37]. One of the strengths of our survey is the large response: more than 100 responses of more than 100 centers worldwide. Therefore, this survey represents international treatment strategies.

Some limitations should be addressed. The absence of a response rate could potentially lead to a bias in the results. We excluded almost half of the responses because they were incomplete or empty, which may have led to selection bias. Although the responses covered six continents, Asia and Africa were less represented. The latter makes sense; since they are not involved in any of the medical networks we have sent the survey to, we have not actively approached countries in these continents. As a result, fewer third-world countries were included in this survey.

We deliberately did not survey the outcomes of the dilatations, i.e., success rate or complications as the outcomes may have been biased by the presence of non-expert centers. Future research, based on the uniform approach we propose in this study, could elaborate on this.

**Fig. 5:**
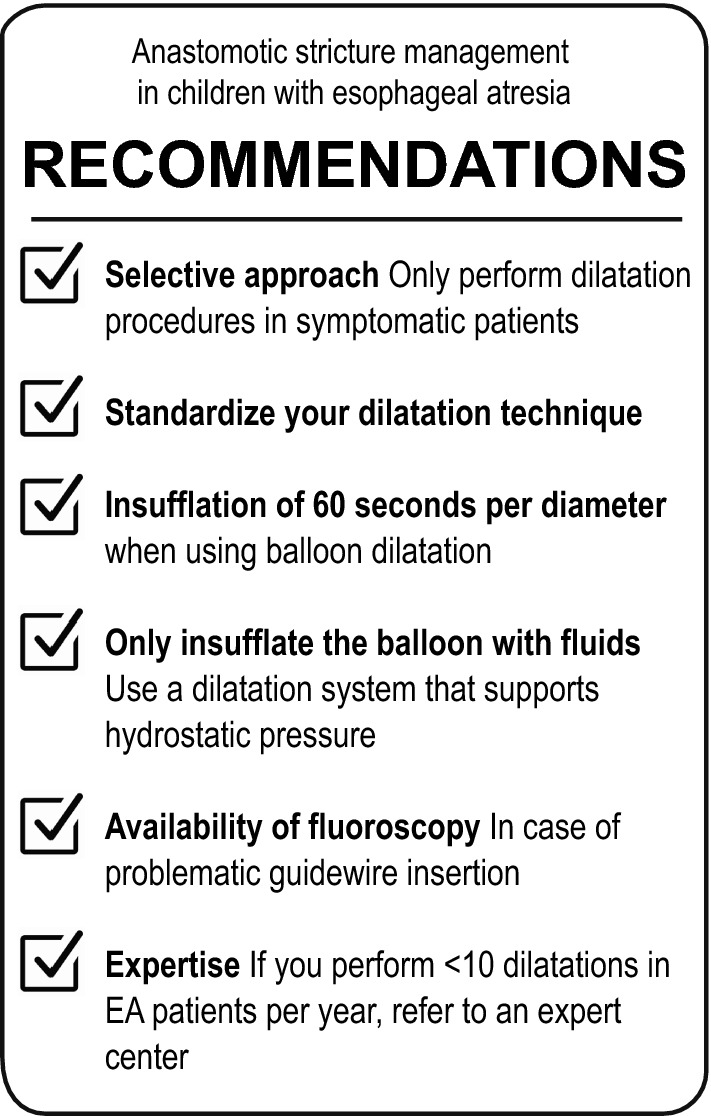
Recommendations for the management of anastomotic strictures in patients with esophageal atresia

In conclusion, this survey confirms the current lack of consensus on the management of anastomotic strictures after EA repair. It emphasizes the importance of harmonizing the approach towards stricture and dilatation management in patients with EA, for which we present several suggestions (see Fig. 5).

As a member of international networks on rare digestive diseases, we strive for optimal patient care for rare inherited and congenital diseases. A systematic and standardized approach is important to improve the clinical standards and patients outcomes, especially in rare diseases where first-level evidence is hard to obtain. In this paper, we extensively discuss the two main dilatation techniques: balloon dilatation and bougienage. The current lack of consent about the choice of dilatation strategy makes it even more important to standardize these two techniques, since this would enable a prospective observational study and possibly a randomized controlled trial in the future.

## Electronic supplementary material

Below is the link to the electronic supplementary material.

Supplementary file1 (PDF 425 kb)
