# Supplementary material for: An international survey on anastomotic stricture management after esophageal atresia repair: considerations and advisory statements
Source: Surg Endosc. 2020 Aug 3;35(7):3653–61. doi: 10.1007/s00464-020-07844-6 (PMC8195894; doi:10.1007/s00464-020-07844-6)
Supplement: Supplementary file 1 — Supplementary file1 (PDF 425 kb) [file 464_2020_7844_MOESM1_ESM.pdf]

Survey Esophageal Dilatation in EA patients

Background:
While survival rates of esophageal atresia have increased to over 93%, anastomotic stricture remains the most frequent postoperative complication (up to 59%). The ESPGHAN guideline states that anastomotic dilation is the first line of therapy for anastomotic strictures, but consensus on the dilatation method that should be used does not exist. A large variety of methods is described in the literature, of which balloon dilatation and bougienage are the most frequently used dilatation methods.

Objectives:
The aim of this survey is to give an overview of the current management of anastomotic strictures by dilatation. The main goal is to give insight in the experience with the different dilatation methods in the different centers worldwide, which could possibly be used in the development of a guideline with consensus of the dilatation method.

Thank you!
Filling out the survey take approximately 10 minutes. Thank you for taking the time to fill out this survey!

Sincerely,

On behalf of the ESPGHAN EA Working Group,
Renato Tambucci & Luigi Dall'Oglio
Bambino Gesù Children's Hospital, Rome, Italy

On behalf of EUPSA,
Chantal ten Kate & Rene Wijnen
Erasmus MC - Sophia Children's Hospital, Rotterdam, the Netherlands

There are 40 questions in this survey

Part A

First, we have some general questions.

To which of the following organizations are you a member? *

Please choose **all** that apply:

- ☐ EUPSA
- ☐ ESPGHAN
- ☐ NASPGHAN
- ☐ AUSPGHAN
- ☐ Other: ________

Multiple answers possible.

What is the name of the center and department you are currently working at? *

Please write your answer(s) here:

Name of the center ________

Name of the department

How many physicians are working in your department? *

Please write your answer here:

What is the medical specialty of the physicians performing endoscopies in your center? Multiple answers possible. *

Please select at least one answer

Please choose **all** that apply:

- ☐ Adult gastroenterologist
- ☐ Adult general surgeon
- ☐ Pediatric gastroenterologist
- ☐ Pediatric surgeon
- ☐ Other:

Do residents or fellows receive training in your center? *

Please choose **only one** of the following:

- ◯ Yes
- ◯ No

Do residents or fellows perform endoscopies? *

Only answer this question if the following conditions are met:
Answer was 'Yes' at question '5 [A5]' (Do residents or fellows receive training in your center?)

Please choose **only one** of the following:

- ◯ Yes
- ◯ No

Part B

Questions 7-11 concern all patients, with or without esophageal atresia.

During a typical month, approximately how many *pediatric endoscopies* (both upper and lower) are performed for any reason (both diagnostic and therapeutic)? *

Please choose **only one** of the following:

- <10
- 10-30
- 31-50
- 51-70
- >70

During a typical month, approximately how many *pediatric upper endoscopies* are performed for any reason (both diagnostic and therapeutic)? *

Please choose **only one** of the following:

- <10
- 10-30
- 31-50
- 51-70
- >70

During a typical month, approximately how many *therapeutic* pediatric upper endoscopies are performed for any reason? *

Please choose **only one** of the following:

- <5
- 5-10
- 11-15
- 16-20
- >20

During a typical month, approximately how many pediatric esophageal dilatation procedures are performed for any reason? *

Please choose **only one** of the following:

- <5
- 5-10
- 11-15
- 16-20
- >20

Which is the technique mainly used to manage esophageal strictures, with focus on balloon or bougie dilatation? *

Please choose **only one** of the following:

- Balloon
- Bougie

- ◯ Both

Part C

The rest of the questionnaire only concerns patients with esophageal atresia (EA).

Overall, how many pediatric patients with EA are currently under follow up in your center? *

Please choose **only one** of the following:

- <20
- 20-40
- 41-60
- 61-80
- 81-100
- >100

Approximately, how many *new* cases of EA are born or referred to your center per year? *

Please choose **only one** of the following:

- ≤5
- 6-10
- 11-20
- >20

Approximately, of the total number of new EA patients, what percentage are long gap EA? *

Please choose **only one** of the following:

- <5%
- 5-10%
- >10%

Approximately, how many dilation procedures are performed per month for anastomotic strictures in EA patients? *

Please choose **only one** of the following:

- <3
- 3-5
- 6-7
- 8-10
- >10

Which is the technique mainly used to manage esophageal anastomotic strictures in EA patients? *

Please choose **only one** of the following:

- Balloon
- Bougie
- Both

If balloon dilatation is the preferred technique, which is the mainly used approach, endoscopic or radiologic? *

Only answer this question if the following conditions are met:
Answer was 'Balloon' *or* 'Both' at question '16 [A16]' (Which is the technique mainly used to manage esophageal anastomotic strictures in EA patients?)

Please choose **only one** of the following:

- ◯ Endoscopic balloon dilatation
- ◯ Radiologically guided balloon dilatation
- ◯ Both

Is a guidewire used routinely? *

Only answer this question if the following conditions are met:
Answer was 'Both' *or* 'Balloon' at question '16 [A16]' (Which is the technique mainly used to manage esophageal anastomotic strictures in EA patients?)

Please choose **only one** of the following:

- ◯ Yes
- ◯ No

Which balloon dilators do you use for the dilatation procedure? *

Only answer this question if the following conditions are met:
Answer was 'Balloon' *or* 'Both' at question '16 [A16]' (Which is the technique mainly used to manage esophageal anastomotic strictures in EA patients?)

Please choose **all** that apply:

- ☐ Rigiflex balloon dilators

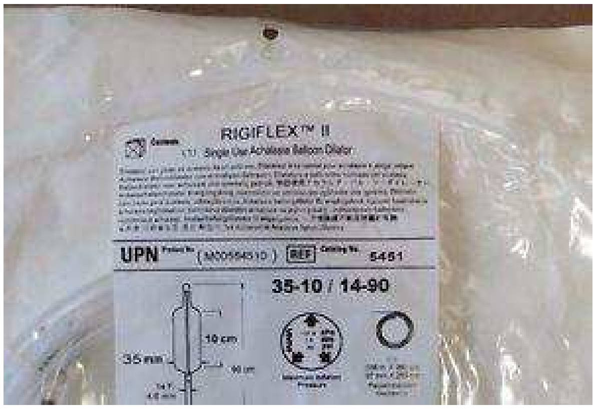

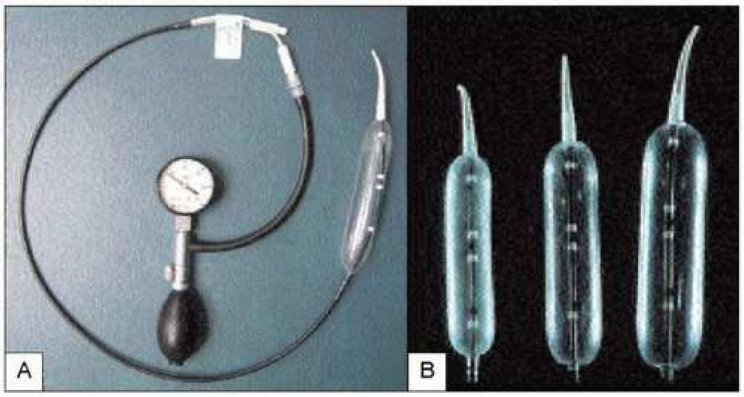

- ☐ Controlled Radial Expension (CRE) balloon dilators

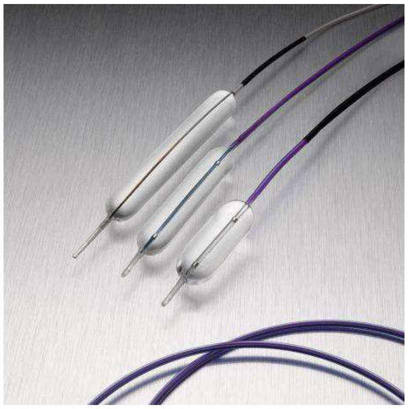

- ☐ Maxforce balloon dilators

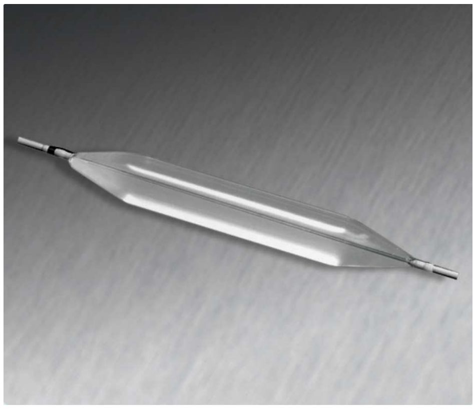

- [ ] VACS balloon dilators

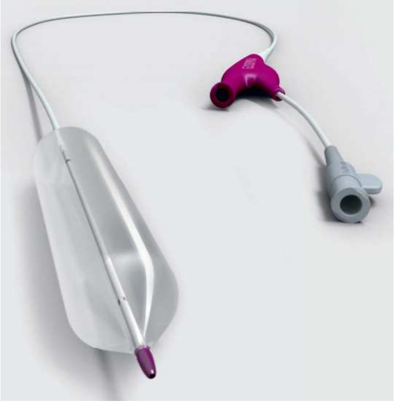

- [ ] Ultra-thin Diamond balloon dilators

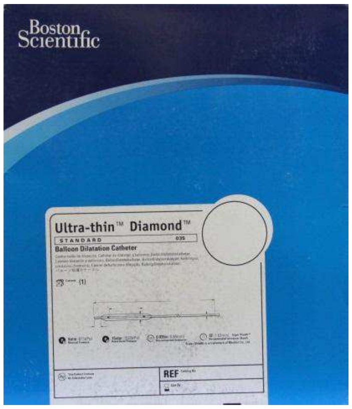

- [ ] Gruentzig-type balloon dilators (Schneider, Medi-tech)
- [ ] I don’t know
- [ ] Other, please specify: ____________

Multiple answers possible.

With which material do you insufflate the balloon? *

Only answer this question if the following conditions are met:
Answer was 'Both' *or* 'Balloon' at question '16 [A16]' (Which is the technique mainly used to manage esophageal anastomotic strictures in EA patients?)

Please choose **all** that apply:

- [ ] Water or natrium chloride

- ☐ Contrast fluid
- ☐ Air
- ☐ Other, please specify:

Is the time of insufflation of the balloon recorded in a standardized protocol? *

Only answer this question if the following conditions are met:
Answer was 'Both' *or* 'Balloon' at question '16 [A16]' (Which is the technique mainly used to manage esophageal anastomotic strictures in EA patients?)

Please choose **only one** of the following:

- ○ Yes
- ○ No

If yes, how many seconds?

Only answer this question if the following conditions are met:
Answer was 'Yes' at question '21 [A21]' (Is the time of insufflation of the balloon recorded in a standardized protocol?)

Please write your answer here:

Which bougie dilators do you use for the dilatation procedure? *

Only answer this question if the following conditions are met:
Answer was 'Bougie' *or* 'Both' at question '16 [A16]' (Which is the technique mainly used to manage esophageal anastomotic strictures in EA patients?)

Please choose **all** that apply:

- ☐ Hurst (blunt-tipped) dilators (non-guidewired)

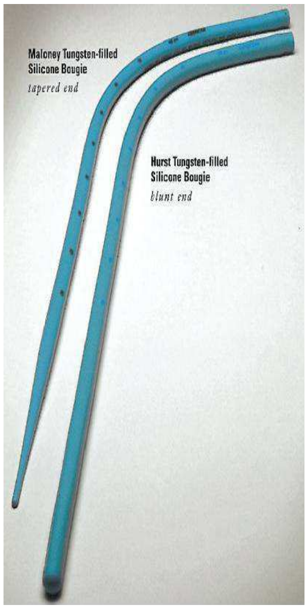

- ☐ Maloney (tapered) dilators (non-guidewired)

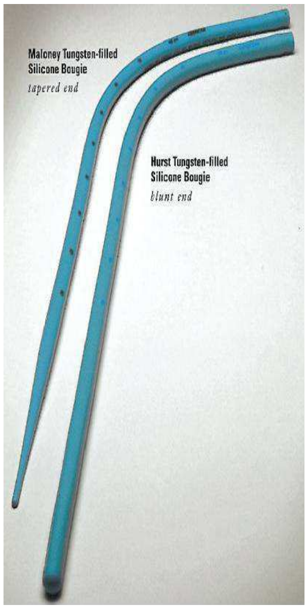

☐ Tucker (rubber) dilators

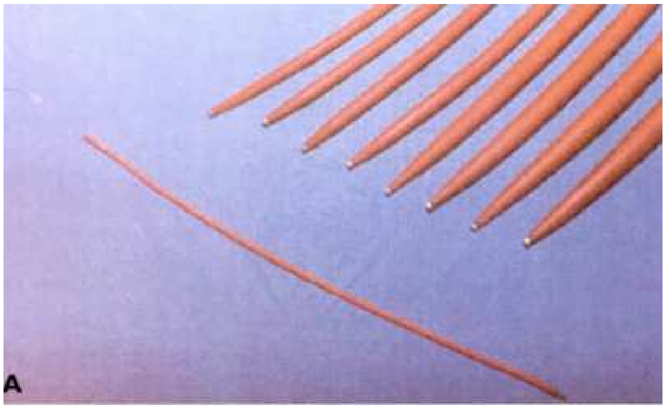

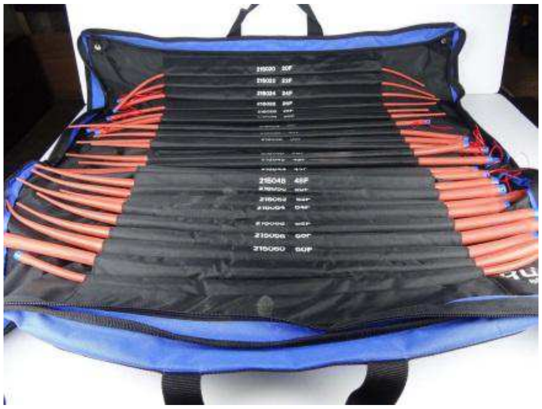

☐ Jackson (silk-woven) dilators

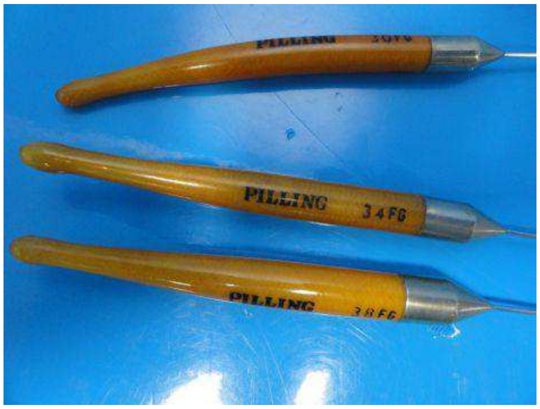

☐ Savary-Gillard dilators

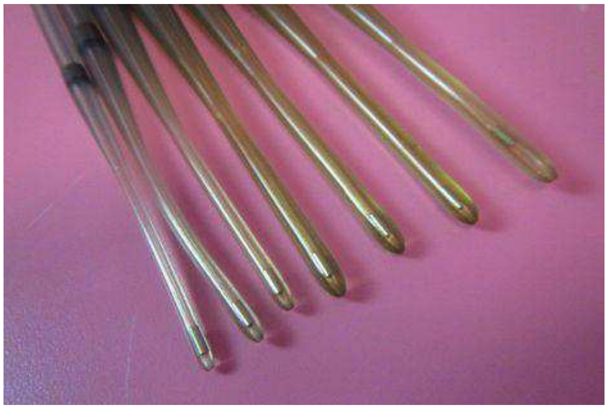

☐ American Dilatation System (Bard)

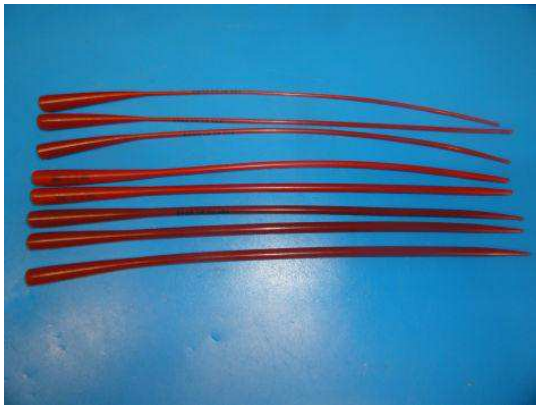

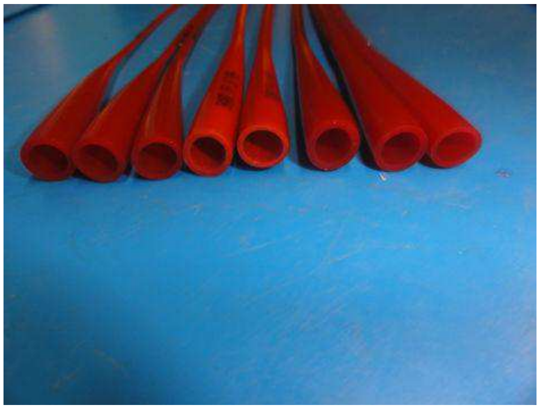

☐ Emerson Teflon dilators

☐ Rehbein (Rush) dilators

☐ I don't know

☐ Other, please specify: ________

Multiple answers possible.

Is the chosen diameter of the bougie dilator determined by a standardized protocol? *

Only answer this question if the following conditions are met:
Answer was 'Bougie' *or* 'Both' at question '16 [A16]' (Which is the technique mainly used to manage esophageal anastomotic strictures in EA patients?)

Please choose **only one** of the following:

◯ Yes

◯ No

If yes, please specify

Only answer this question if the following conditions are met:
Answer was 'Yes' at question '24 [A23]' (Is the chosen diameter of the bougie dilator determined by a standardized protocol?)

Please write your answer here:

Approximately, how many cases per year experience complications after dilatation (e.g. perforation, hemorrhage)? *

Please choose **only one** of the following:

- ◯ <3
- ◯ 4-5
- ◯ 6-7
- ◯ 8-10
- ◯ >10

Which is the preferred approach used to manage esophageal anastomotic strictures in EA patients? *

Please choose **only one** of the following:

- ◯ Routine dilatations (planned in advance, to prevent symptoms)
- ◯ Selective dilatations (only in symptomatic patients)

In case of recurrent and refractory esophageal anastomotic strictures, which adjunctive treatments are available at your center? *

Please choose **all** that apply:

- ☐ Local injection of steroids
- ☐ Topical application of mitomycin C
- ☐ Esophageal stenting
- ☐ Incisional therapy
- ☐ Other:

Multiple answers possible.

Which is the preferred first-line adjunctive treatment in use at your center? *

Please choose **all** that apply:

- ☐ Local injection of steroids
- ☐ Topical application of mitomycin C
- ☐ Esophageal stenting
- ☐ Incisional therapy
- ☐ Other:

Approximately, how many cases per year undergo surgery due to failure of conservative managements for anastomotic strictures? *

Please choose **only one** of the following:

- ◯ 0-1
- ◯ 1-3
- ◯ 4-5

- [ ] >5

Part D

These last questions give us an overview of the number of patients treated in each hospital. You can aswer all questions approximately. If you don't know, please fill in 'unknown'.

How many EA patients underwent an endoscopy in your center in 2017? *

Please write your answer here:

How many EA patients were treated for long gap EA in your center in 2017? *

Please write your answer here:

How many EA patients underwent at least one esophageal dilatation procedure due to an anastomotic stricture in your center in 2017? *

Please write your answer here:

How many EA patients experienced complications after a dilatation procedure in your center in 2017? *

Please write your answer here:

How many EA patients experienced *recurrent* anastomotic strictures (minimum of 3 dilatations needed) in your center in 2017? *

Please write your answer here:

How many EA patients experienced *refractory* anastomotic strictures (minimum of 5 dilatations needed) in your center in 2017? *

Please write your answer here:

How many EA patients underwent surgery due to a refractory anastomotic stricture in your center in 2017? *

Please write your answer here:

Part E

Finally, we would like to make an inventory of potential centers to collaborate with in future studies on this subject.

Would your center be interested to participate in a prospective (observational) study, supported by the ESPGHAN EA Working Group, towards the efficacy of balloon dilatation and bougienage in EA patients? If yes, you will be sent information about the planned study without any further obligation by email.

*

Please choose **only one** of the following:

- ◯ Yes
- ◯ No

What is your email address? *

Only answer this question if the following conditions are met:
Answer was 'Yes' at question '38 [A36]' (Would your center be interested to participate in a prospective (observational) study, supported by the ESPGHAN EA Working Group, towards the efficacy of balloon dilatation and bougienage in EA patients? If yes, you will be sent information about the planned study without any further obligation by email.)

Please write your answer here:

Do you have any further comments on this survey?

Please write your answer here:

Submit your survey.
Thank you for completing this survey.
